# Multispectral optoacoustic tomography is more sensitive than micro-computed tomography for tracking gold nanorod labelled mesenchymal stromal cells

**DOI:** 10.1002/jbio.202300109

**Published:** 2023-07-23

**Authors:** Alejandra Hernandez Pichardo, James Littlewood, Arthur Taylor, Bettina Wilm, Raphaël Lévy, Patricia Murray

**Affiliations:** 1 Department of Molecular Physiology and Cell Signalling, Institute of Systems, Molecular and Integrative Biology, University of Liverpool, Liverpool, UK; 2 Centre for Pre-clinical Imaging, University of Liverpool, Liverpool, UK; 3 iThera Medical GmbH, Munich, Germany; 4 Université Sorbonne Paris Nord and Université de Paris, INSERM, LVTS, Paris, France

**Keywords:** bioluminescence, cell tracking, gold nanorods, mesenchymal stromal cells, micro-CT, multispectral optoacoustic tomography

## Abstract

Tracking the fate of therapeutic cell types is important for assessing their safety and efficacy. Bioluminescence imaging (BLI) is an effective cell tracking technique, but poor spatial resolution means it has limited ability to precisely map cells in vivo in 3D. This can be overcome by using a bimodal imaging approach that combines BLI with a technique capable of generating high-resolution images. Here we compared the effectiveness of combining either multispectral optoacoustic tomography (MSOT) or micro-computed tomography (micro-CT) with BLI for tracking the fate of luciferase^+^ human mesenchymal stromal cells (MSCs) labelled with gold nanorods. Following subcutaneous administration in mice, the MSCs could be readily detected with MSOT but not with micro-CT. We conclude that MSOT is more sensitive than micro-CT for tracking gold nanorod-labelled cells in vivo and depending on the route of administration, can be used effectively with BLI to track MSC fate in mice.

**Figure:**
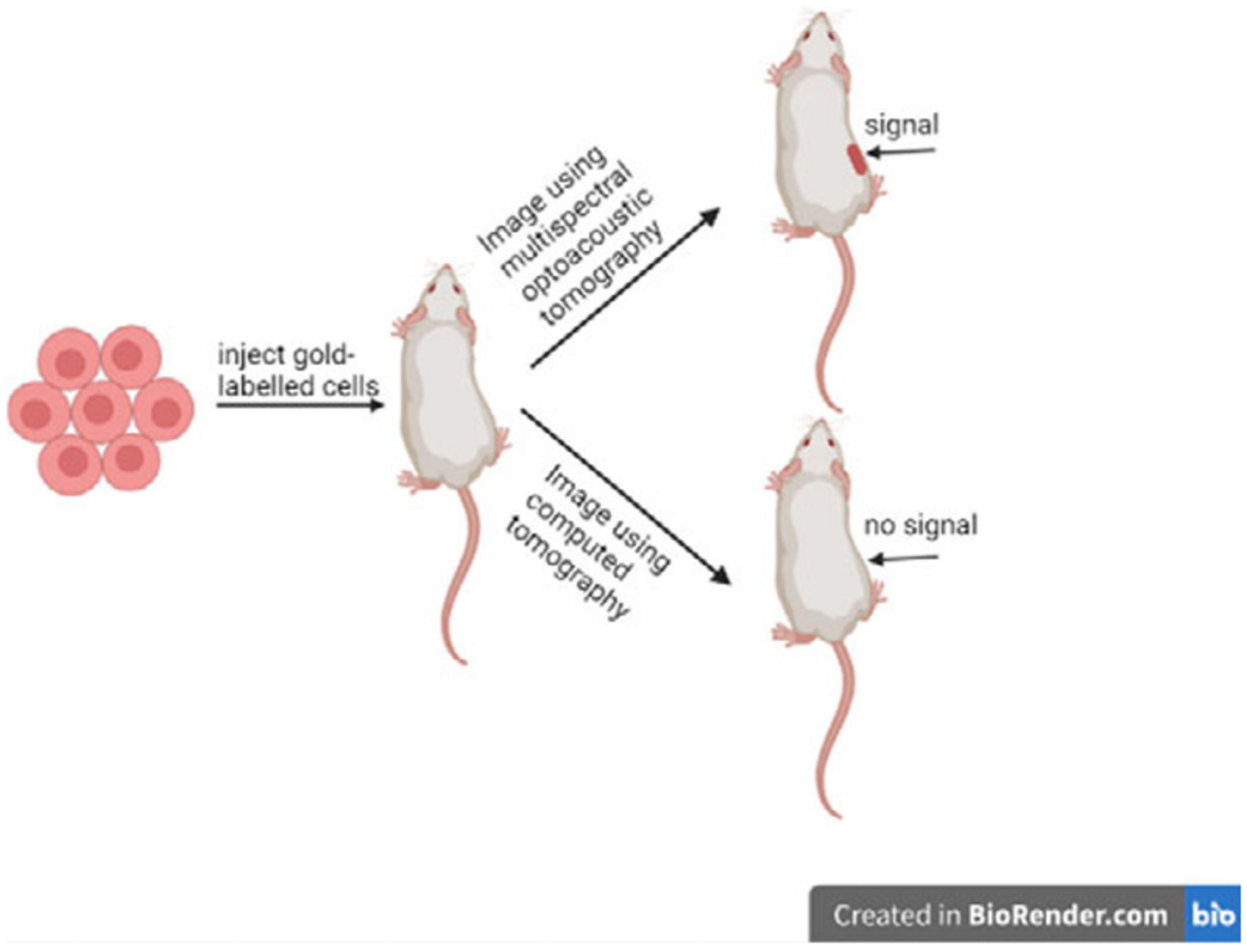

## 1 Introduction

The ability to image cells and non-invasively track their fate in animal models has become increasingly important in assessing the long-term safety and efficacy of cell-based regenerative therapies. Moreover, the ability to monitor the biodistribution of cells over time can also offer key insights into their mechanisms of action; for instance, establishing whether engraftment in the target organ is required for the cells to have any beneficial effects.

The intravenous administration of mesenchymal stromal cells (MSCs) in mice leads to their entrapment in the pulmonary vasculature and inability to reach other organs [1–3]. Bioluminescence imaging (BLI) has been key in this discovery. BLI is a non-invasive, whole-animal, pre-clinical imaging modality with high sensitivity and a temporal resolution of seconds to minutes [4]. BLI allows longitudinal cell tracking via a reporter gene encoding luciferase, an enzyme that oxidises a substrate to generate light [5, 6]. However, BLI is limited by low spatial resolution (3–5 mm) which does not allow the biodistribution of the cells to be mapped at the intra-organ level [7].

Multispectral optoacoustic tomography (MSOT) is a non-invasive imaging modality that provides functional and anatomical information in real-time. MSOT operates by the photoacoustic effect: incident modulated light energy is absorbed leading to thermo-elastic expansion and the generation of ultrasound waves [8]. MSOT uses a range of near-infrared excitation wavelengths, and subsequent spectral unmixing algorithms allow the identification of the optical signatures of endogenous and administered contrast agents. It benefits from high spatial (100 μm) and temporal (0.1 s) resolutions. Its main limitation for tracking cells delivered intravenously is that due to the high air content of the lungs and the behaviour of sound in this medium, MSOT is unable to image this organ.

Computed tomography (CT) is inherently effective for lung imaging due to the native contrast provided by the airspaces. Micro-CT is a non-invasive imaging modality that generates 3D anatomical images based on the differential x-ray attenuation of materials [9]. Although efforts are being made to reduce the radiation doses in clinical CT scans, this remains a problem when undertaking longitudinal imaging in small animals [10, 11]. The advantages of CT include high spatial resolution (50–200 μm) with high signal-to-noise ratio, high depth of penetration, quantitative capabilities, high temporal resolution, and cost-effectiveness, making it a widely applied imaging modality in the clinic [10].

To distinguish administered cells from endogenous tissue in animal models, cells need to be labelled with a contrast agent. Gold has been an extensively studied element for this purpose since its high atomic number gives it the ability to produce higher contrast than iodine, the current gold standard for CT imaging [12]. Moreover, gold nanoparticles are easily synthesised and functionalised [13]. In particular, gold nanorods (GNRs) are a suitable contrast agent for both micro-CT and MSOT. The micro-CT contrast is due to gold’s high density and atomic number [9] whilst MSOT contrast can be achieved via the near-infrared (NIR) longitudinal surface plasmon bands that are characteristic of GNRs [14]. Cell labelling with GNRs is achieved by endocytosis. The tight packing of the GNRs within endosomes can result in the GNRs undergoing plasmon coupling, altering their optical properties and compromising MSOT detection [15]. Coating the GNRs with silica prevents plasmon coupling and does not affect cell viability, allowing MSOT to reach its full potential [16].

Our group has previously applied a dual BLI/MSOT imaging strategy to track GNR-labelled cells [15, 17]. Here, the possibility of expanding this approach to include micro-CT is explored. In addition, we aimed to compare the effectiveness of MSOT and micro-CT for tracking cells labelled with silica-coated GNRs to study the in vivo biodistribution of MSCs delivered subcutaneously or intravenously.

## 2 Methods

### 2.1 Gold nanorod characterisation

Commercially available silica-coated gold nanorods (GNRs) pre-adsorbed with bovine serum albumin (BSA) were purchased from Creative Diagnostics (2.5 mg/mL). Their properties were characterised using transmission electron microscopy (TEM) (Tecnai G2 Spirit BioTWIN) coupled to a Gatan RIO16 camera and Vis–NIR spectroscopy (FLUOstar Omega, BMG Labtech). GNR stability was studied by incubating the GNRs in cell culture medium at 37°C in a humidified incubator, with 5% CO_2_. After 24 h, the GNRs were recovered by centrifugation at 13,000 × *g* for 20 min, washed three times with dH_2_O and imaged by TEM. Particles were deposited onto glow discharged fomvar/carbon coated grids for 10 min, excess wicked off and stained for 20 s with 1% aqueous uranyl acetate.

### 2.2 Cell isolation, generation of reporter cell line and culture

Human umbilical cord-derived mesenchymal stromal cells (hUC-MSCs) were obtained from the National Health Service Blood and Transplant (NHSBT, UK) at passage 3 (p3). The hUC-MSCs were transduced with a lentiviral vector encoding luc2 firefly luciferase (FLuc) reporter under the constitutive elongation factor 1-α (EF1α) promoter and the ZsGreen fluorescent protein downstream of the bioluminescence reporter via an IRES linker. The pHIV-Luc2-ZsGreen vector was kindly gifted by Bryan Welm and Zena Werb (Addgene plasmid #39196) [18]. To obtain a >98% FLuc positive population, the cells were sorted based on ZsGreen fluorescence.

The cells were grown in MEM-α and supplemented with 10% foetal bovine serum (FBS) (Gibco) and kept at 37°C in a humidified incubator, with 5% CO_2_.

### 2.3 Cell viability assay

5 × 10^3^ cells were seeded into 96-well plates (Corning) and allowed to attach for 24 h. The viability of hUC-MSCs after 24 h exposure to increasing concentrations of GNRs was determined by the CellTiter-Glo™ Luminescent Cell Viability Assay (Promega Corporation), which generates luminescent signals based on ATP levels. Tests were performed in triplicate with two PBS washing steps between GNR exposure and the assay. Luminescence was measured in a multi-well plate reader (FLUOstar Omega, BMG Labtech).

### 2.4 Assessing the extent of GNR uptake by hUC-MSCs

hUC-MSCs were seeded at 13 × 10^3^ cells/cm^2^ into 24-well plates (Corning) and allowed to attach for 24 h. Based on the available material, cells were exposed to 1:100 and 1:10 GNR dilutions (0.125 or 0.25 mg/mL) in cell culture medium for 24 h. After this period, the cells were washed with PBS to remove excess GNRs and fixed with paraformaldehyde (4%, w/v in PBS, pH 7) for 20 min at room temperature (RT). GNR uptake by the cells was assessed by using a silver enhancement solution kit (Sigma SE100) according to the manufacturer’s instructions. After rinsing three times with PBS, the cells were imaged by light microscopy with a Leica DM IL microscope coupled to a DFC420C camera.

### 2.5 Ethics statement

All animal procedures were performed under a licence granted under the Animals (Scientific Procedures) Act 1986 and were approved by the University of Liverpool Animal Welfare and Ethics Review Board (AWERB).

### 2.6 Animal experiments

Eight- to ten-week-old female albino (C57BL/6) (B6N-TyrC-Brd/BrdCrCrl, originally received from the Jackson Lab) mice were used for all animal experiments. Mice were housed in individually ventilated cages (IVCs) under a 12-h light/dark cycle and provided with standard food and water ad libitum.

Mice were injected with 5 × 10^5^ FLuc-hUC-MSCs (hUC-MSCs hereinafter) suspended in 100 μL of PBS by either intravenous (IV) or subcutaneous (SC) administration to the flank, and subsequently imaged via BLI, MSOT and micro-CT, all under terminal anaesthesia with isoflurane. At the end of the experiment, the animals were culled by cervical dislocation and all efforts were made to prevent suffering.

### 2.7 Bioluminescence imaging

Immediately after cell injection, the animals received a SC injection of D-Luciferin (10 μL/g [body weight] of a 47 mM stock solution). 20 min later, the animals were imaged with an IVIS Spectrum instrument (Perkin Elmer). All data is displayed in radiance (photons/second/centimetre [2]/steradian), where the signal intensity scale is normalised to the acquisition conditions.

### 2.8 Multispectral optoacoustic tomography imaging

All imaging was performed in the inVision 256-TF MSOT imaging system (iThera Medical, Munich, Germany). Tissue-mimicking imaging phantoms with a 2 cm diameter were constructed from 1.5% (w/v) agar and 0.4% (w/v) intralipid in distilled water [19]. Two cavities were created to facilitate insertion of clear straws containing either unlabelled hUC-MSCs or hUC-MSCs labelled with 0.25 mg/mL GNRs. 5 × 10^5^ hUC-MSCs were prepared by labelling and trypsinisation as described above and suspended in 100 μL of PBS. Then, the whole volume was inserted into the phantom cavity.

The agar phantoms with inserts were imaged at 61 wavelengths (680–980 nm in 5 nm steps) at 25°C. Three frames were measured per wavelength and averaged.

To image mice in vivo, the torso was shaved and deepilated using Veet Hair removal cream (Reckitt Benckiser, UK) 24 h before imaging. Mice were imaged at 34°C. In mice receiving subcutaneous hUC-MSC injection, scans were acquired at the site of injection at 61 wavelengths (680–980 nm in 5 nm steps) in 1 mm slices. Ten frames per wavelength were measured and averaged. In mice receiving intravenous hUC-MSC injection, scans were acquired at the lungs at 61 wavelengths (680–980 nm in 5 nm steps) in 1 mm slices. Additionally, images were acquired through the full volume of all animals at eight wavelengths (660, 700, 730, 750, 760, 800, 850, 900 nm) in 1 mm slices.

For image processing, the ViewMSOT 4.0.1.34 (iThera Medical, Germany) was used. Data were reconstructed with the back-projection algorithm. Multispectral unmixing was performed using the linear regression algorithm. Images were unmixed for haemoglobin, oxyhaemoglobin, melanin, and the GNR MSOT spectrum.

### 2.9 Micro-computed tomography

Agar phantoms with inserts, as used for MSOT, were imaged using an aluminium filter 0.5 mm thick or a 0.06 mm copper filter with an applied x-ray tube voltage of 90 kV in a Quantum GX micro-CT (Rigaku Corporation). Images were acquired with a field of view (FOV) of 25 mm giving a voxel size of 50 μm.

After MSOT imaging, the mice were culled by cervical dislocation and their carcasses were imaged using an aluminium filter 0.5 mm thick and an applied x-ray tube voltage of 90 kV with the same instrument. Images were acquired with a FOV of 25 mm giving a voxel size of 50 μm. Surface-rendered 3D models were constructed for 3D viewing of the analysed mice. Volume rendered 3D images were generated using the Quantum GX software version 3.0.39.5100.

### 2.10 Statistical analysis

All values in graphs are represented as mean ± standard deviation. The statistical analysis was performed using the GraphPad Prism 8.4.2 software. The type of statistical test and the number of replicates included in the analyses are indicated in the figure legends.

## 3 Results

### 3.1 Gold nanorod characterisation

**Figure 1:**
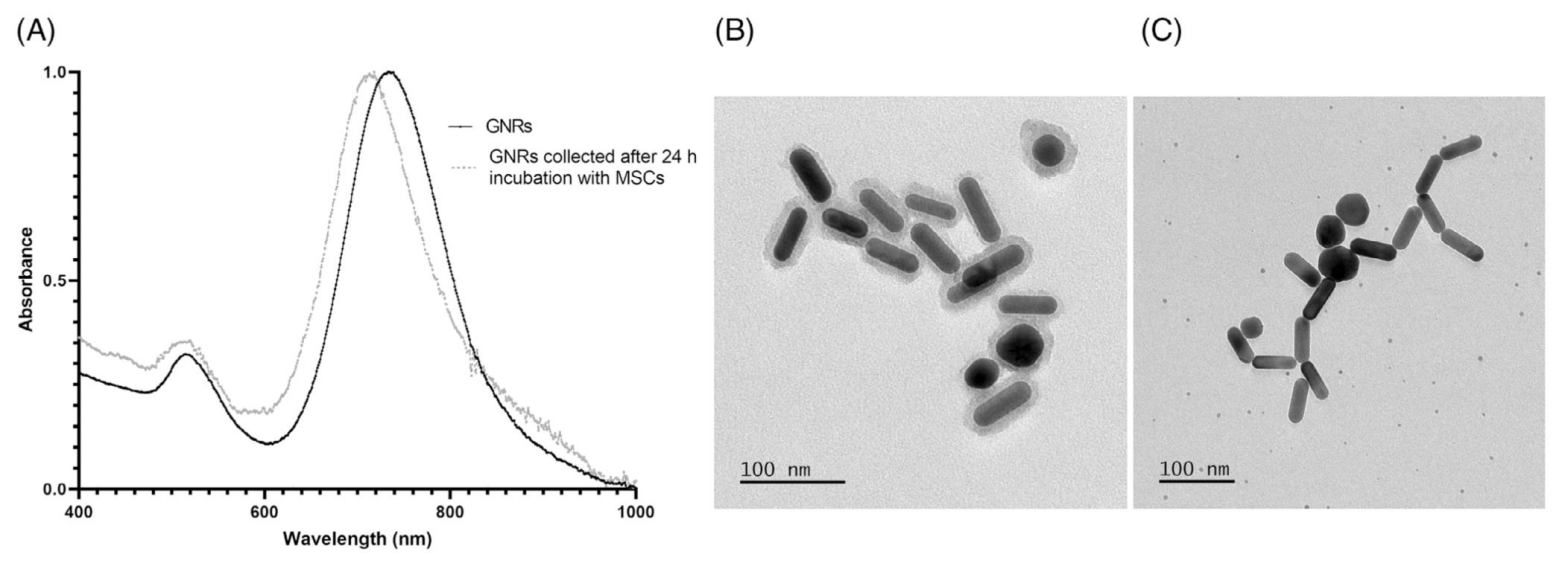
Characterisation of GNRs. (A) Vis–NIR spectrum of GNRs. (B) Representative TEM picture of silica-coated GNRs. (C) TEM image of GNRs 24 h post incubation in cell culture medium.

To characterise the silica-coated GNRs, their absorbance spectrum was assessed using Vis–NIR spectroscopy which revealed that their longitudinal surface plasmon resonance (LSPR) peaks at 738 nm. The integrity of the silica shell was evaluated after incubation in cell medium as etching might occur [20]. After 24 h, the GNRs lose the silica coating resulting in a 25 nm LSPR left shift with a peak at 713 nm (Figure 1A).

The size of the GNRs and the thickness of the silica shell was determined using transmission electron microscopy (TEM). The core size was 55.77 ± 7.32 nm length by 17.36 ± 1.99 nm width with a silica shell thickness of 7.25 ± 1.65 nm. TEM confirmed the loss of the silica shell after incubation in cell medium (Figure 1B,C). Despite the LSPR shift, the absorbance of the GNRs remained within the optical window (700–900 nm) where endogenous light absorbance of biological tissues is lower, making them good candidates for cell labelling [21].

### 3.2 Gold labelling of human umbilical cord mesenchymal stromal cells

**Figure 2:**
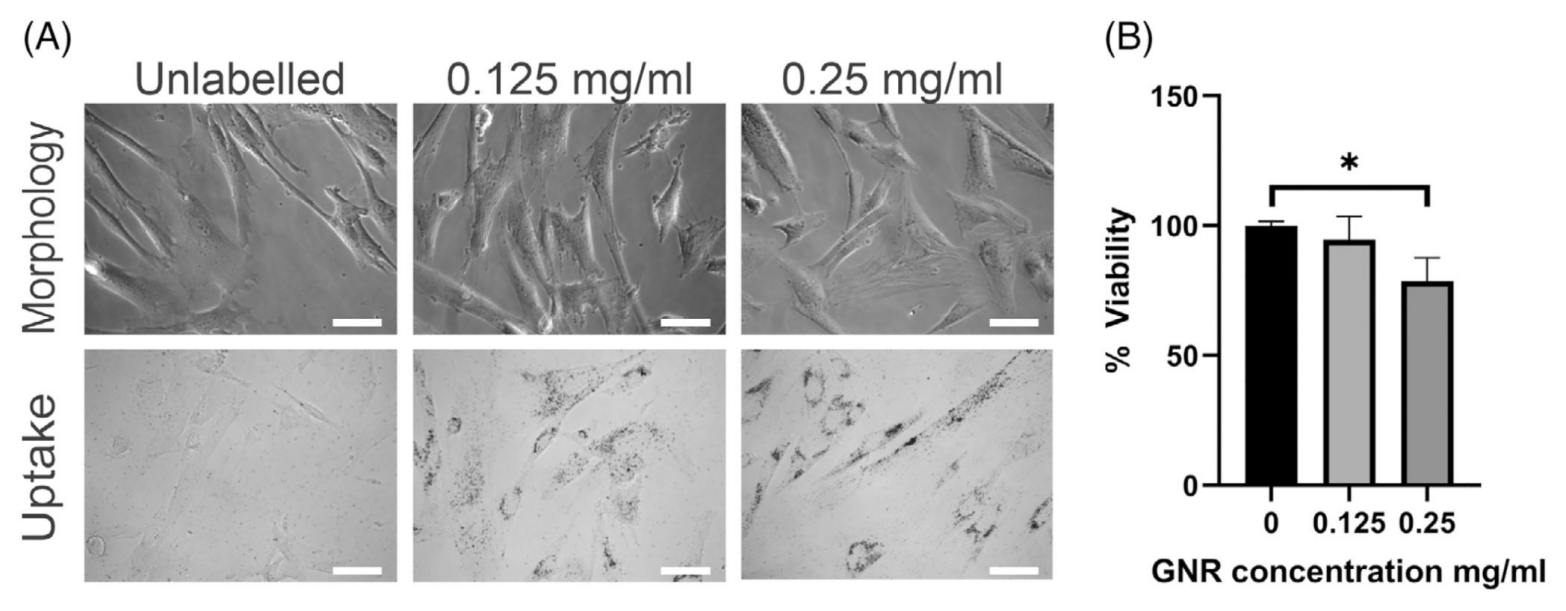
Cell morphology, viability and nanorod uptake after GNR labelling. (A) Cell morphology (top) after labelling with GNRs at different concentrations and uptake (bottom) assessed by silver staining. Optical microscopy images of hUC-MSCs labelled with different GNR concentrations for 24 h. Dark contrast is generated by silver-enhanced staining of GNRs. Scale bar 100 μm. (B) Cell viability. One-way ANOVA with Tukey’s multiple comparisons *p < 0.0141. *n* = 3.

Next, we assessed the effect of different GNR concentrations on morphology, labelling efficiency and cell viability. No overt changes in cell morphology were observed via microscopy 24 h after GNR labelling (Figure 2A, top). Using the gold-specific silver staining, GNR uptake by the hUC-MSCs was confirmed at all concentrations, showing a clear dose-dependent uptake trend as indicated by an increase in contrast. The gold particles accumulated in the perinuclear space, consistent with lysosomal accumulation as previously reported (Figure 2A, bottom) [22, 23].

To determine cell viability, we quantified the total amount of ATP in cells labelled for 24 h with 0.125 mg/mL or 0.25 mg/mL GNRs. Our results indicated that viability levels were at 94.6%, and 78.7% of unlabelled control cells. Whilst a significant reduction in viability was observed at the highest concentration, the GNRs were not overtly toxic to the cells (Figure 2B). Given that labelling with 0.25 mg/mL yielded more uptake (Figure 2A, bottom right), this concentration was taken forward for cell phantom imaging with MSOT and micro-CT.

### 3.3 MSOT/CT imaging of GNR labelled hUC-MSCs in phantoms

**Figure 3:**
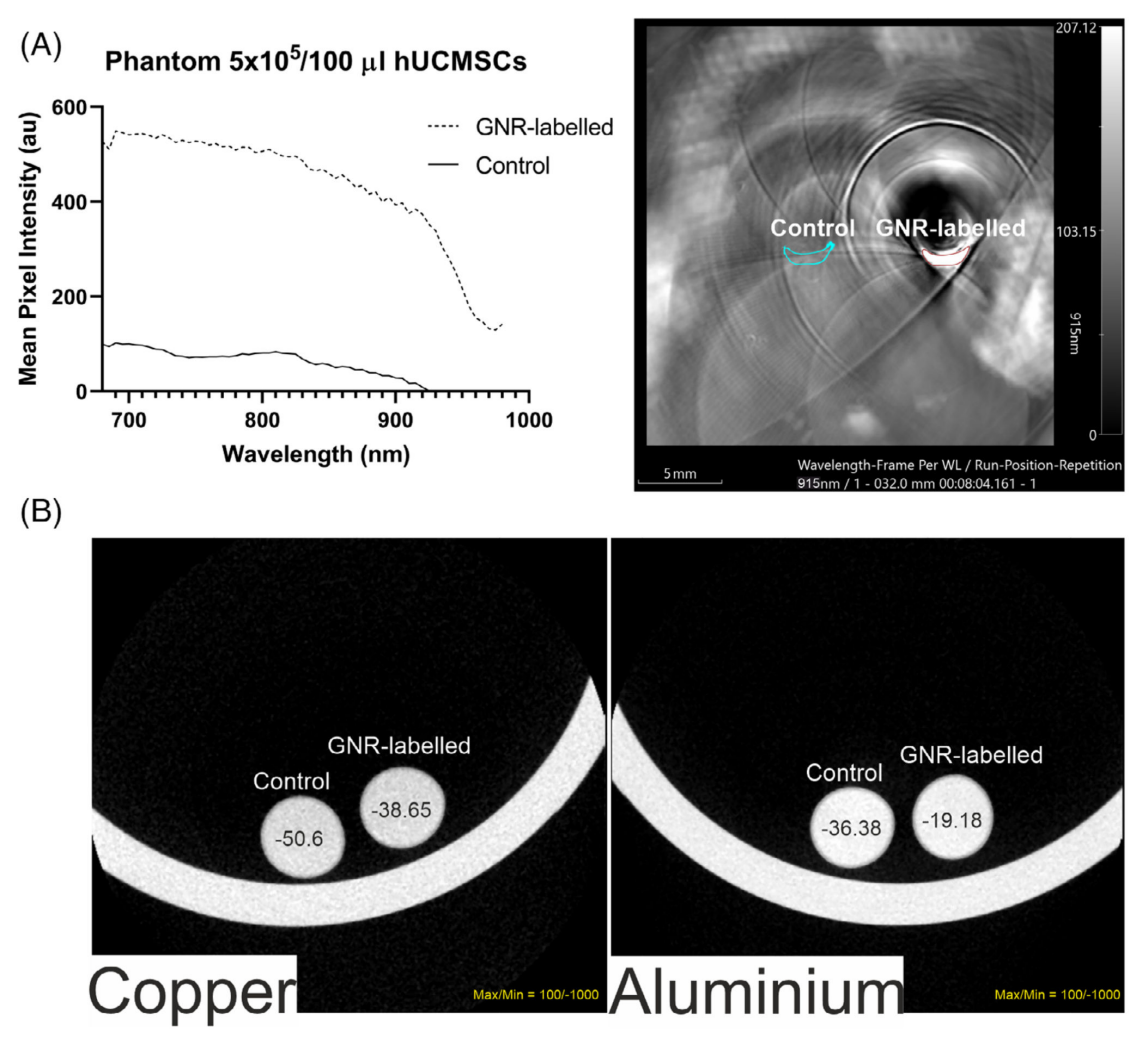
Phantom imaging of GNR-labelled and unlabelled hUC-MSCs. (A) MSOT demonstrates a clear distinction in signal intensity between samples containing labelled or unlabelled cells. The left panel shows the spectrum of GNR-labelled and unlabelled MSCs. The right panel shows a maximum intensity projection of imaging phantoms containing MSCs. (B) Micro-CT fails to detect GNR-labelled MSCs, with both samples having similarly low contrast regardless of the imaging filter used. Copper (left); aluminium (right). HU scale max/min = 100/−1000. Calculated HU values are shown within the corresponding samples.

Before in vivo imaging, it is important to establish whether the GNR-labelled MSCs can be visualised by MSOT and micro-CT. To do this, 5 × 10^5^ hUC-MSCs were suspended in 100 μL PBS (GNR-labelled and control MSCs) into an agar phantom and MSOT intensity was recorded at wavelengths ranging from 680 to 980 nm. The absorbance spectrum of the labelled hUC-MSCs measured with the MSOT instrument was broadened compared to the spectrum of control hUC-MSCs (Figure 3A, left) [15]. Nevertheless, labelled cells could still be detected after applying a multispectral unmixing algorithm, where they are seen as a crescent shape due to the cells sedimenting to the bottom of the phantom (Figure 3A, right). Imaging of the phantom by micro-CT showed no difference in contrast between unlabelled and GNR-labelled MSCs when using either the standard filter (aluminium) or a specialised copper filter for the detection of metals (Figure 3B). Moreover, we imaged nanoparticle suspensions at concentrations ranging from 1 to 80 mM and observed minimal contrast generation at the highest concentration (Figure S1).

### 3.4 In vivo multi-modal monitoring of GNR-labelled cells administered subcutaneously or intravenously

To further investigate the potential for cell tracking of gold-labelled MSCs by MSOT and micro-CT, 5 × 10^5^ control hUC-MSCs cells or hUC-MSCs labelled with 0.25 mg/mL GNRs were administered either IV or SC.

**Figure 4:**
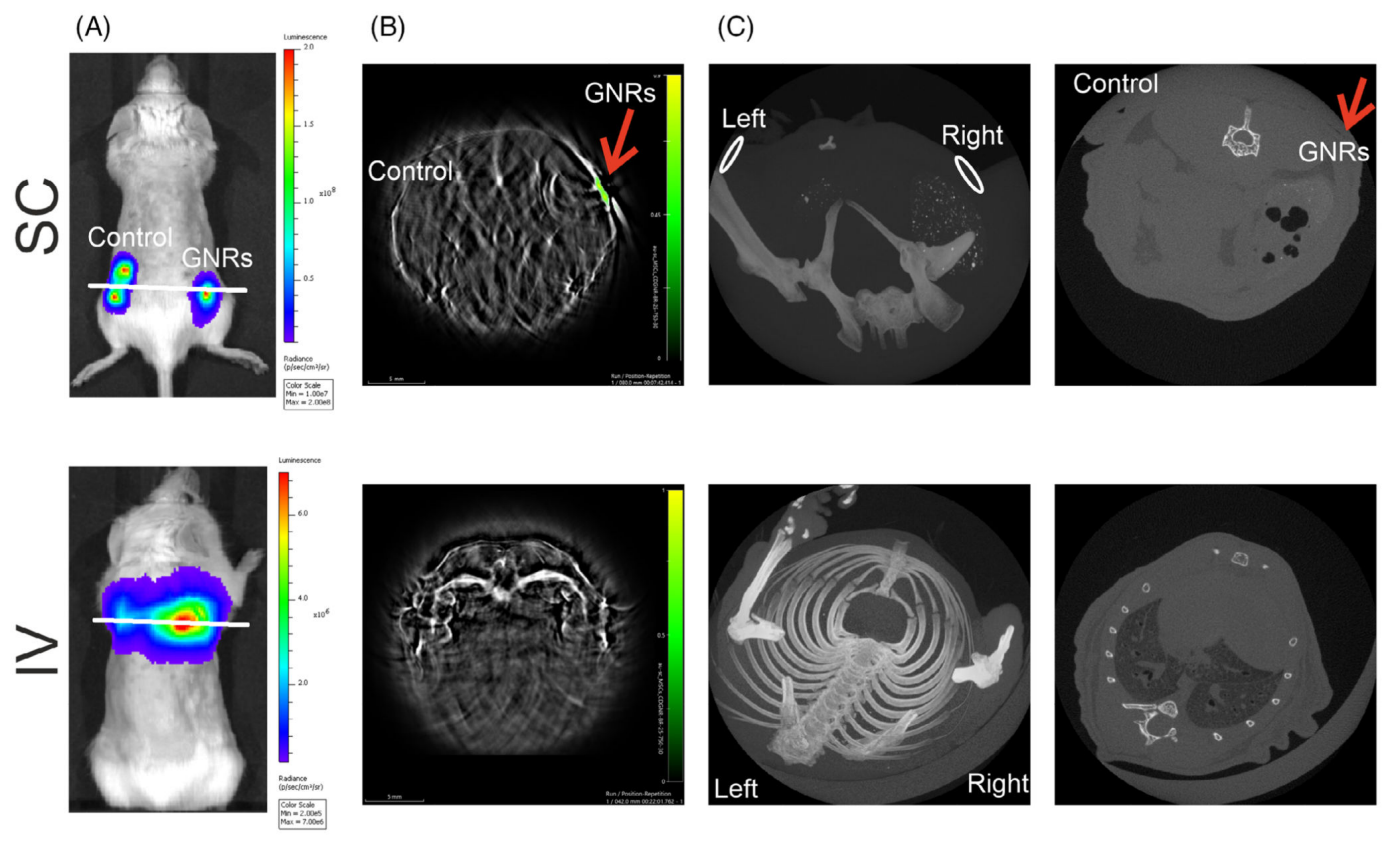
Representative bioluminescence, MSOT and micro-CT imaging of mice after receiving hUC-MSCs. Top row shows representative images of a mouse following SC administration of hUC-MSCs imaged with BLI (A), MSOT (B) and micro-CT (C). Bottom row shows representative images of a mouse following IV administration of hUC-MSCs imaged with BLI (A), MSOT (B) and micro-CT (C). The BLI images are planar, whereas MSOT and micro-CT generate 3D tomographic images. The position of the white line on the BLI images indicates the optical MSOT sections shown in (B) and the optical micro-CT sections shown in the right panels of (C). (A) BLI shows that cells injected SC remain at the site of injection whereas when injected IV, they localise to the lungs. (B) GNR-labelled cells were distinguished from any internal organ when injected SC (green scale displays GNR-specific signal, indicated by arrow) but not IV. (C) Micro-CT fails to generate contrast of GNR labelled cells administered via either route. 3D volume rendered images (left), site of cell location is indicated by ovals. 2D representative micro-CT section of the site of SC injection (top, right; indicated by a red arrow) and lungs (bottom, right). HU window Max/min = 1000/−500. *n* = 6.

BLI demonstrated that following either delivery route, the hUC-MSCs showed a strong luminescent signal confirming their presence in the mice. SC injection resulted in the cells localising at the site of injection, with comparable signals at the site of unlabelled (left flank) or gold-labelled cells (right flank). By contrast, when the cells were administered IV, the hUC-MSCs localised to the lungs (Figure 4A).

MSOT confirmed the presence of GNR-labelled cells in the mouse right flank as observed by the high contrast resulting from the GNRs. On the other hand, the unlabelled cells failed to generate optoacoustic contrast, demonstrating the ability of MSOT to detect gold-labelled cells. As expected, the GNR-labelled cells in the lungs could not be detected due to the high air content within this organ (Figure 4B).

Finally, the SC injected GNR-labelled hUC-MSCs could not be detected by micro-CT. A clear image of the lungs could be obtained by micro-CT but the GNR-labelled MSCs were undetectable (Figure 4C).

These results demonstrate that 5 × 10^5^ luciferase-expressing hUC-MSCs can be detected by BLI following both IV and SC administration, gold-labelled hUC-MSCs can be detected by MSOT following SC administration, but micro-CT lacks the sensitivity to detect the hUC-MSCs via either administration route.

## 4 Discussion

One aim of multi-modal imaging strategies is the analysis of the whole-body and intra-organ biodistribution of administered cells in preclinical animal models. In this study, we explored the feasibility of combining the sensitivity of BLI with the spatial resolution of MSOT and the ability to image the lungs by micro-CT to track GNR-labelled MSCs in vivo.

Photoacoustic imaging uses contrast that can be endogenous (e.g. due to absorption by haemoglobin) or exogenous via the use of contrast agents [15]. For cell tracking by MSOT, labelling with gold nanorods has been a method of choice as these particles have a longitudinal plasmon band with strong absorption in the near-infrared [15, 24–26]. Therefore, the silica-coated GNRs used in this study, with LSPR bands at 738 nm or 713 nm in cell medium are good candidates for generating contrast in cells.

Previously, our group showed that a BLI/MSOT strategy to monitor intracardially administered GNR-labelled FLuc + mouse MSCs revealed the presence of cells in the head, liver and kidneys of mice with both imaging techniques, proving the efficacy of this methodology for cell tracking [17].

Here, in line with earlier publications, BLI showed that IV injection leads to hUC-MSC accumulation and trapping in the lungs [1–3]. Due to differences in sound propagation in air, the capacity of MSOT to detect GNR-labelled cells within the lung is hampered. However, following subcutaneous injection, GNR-labelled hUC-MSCs could be detected by MSOT.

Micro-CT has been used to track gold-labelled cells in the lung, as well as in other organs in vivo and ex vivo. Cell labelling strategies to achieve this vary widely, including the use of gold nanoparticles with different surface chemistries, diameters, initial gold concentrations, and incubation times [27–38] (Table 1). It has been shown that the properties of gold nanoparticles impact cell uptake [27], which agrees with the variation in uptake efficiency reported in these studies. Moreover, cell number, administration routes and injection volumes, tracking time, micro-CT scanners, and scanning settings differ greatly between studies. This reflects the complexity of comparing results in the field of gold-labelled cell tracking by micro-CT. Nonetheless, at least 11 reports suggest that micro-CT enables longitudinal tracking of gold-labelled cells [27–38], though in seven of these, the number of cells administered was considerably higher than in our study (i.e. between 1 × 10^6^ and 4 × 10^6^) (Table 1).

In contrast, despite BLI and MSOT confirming the presence of cells in the mice, our study failed to detect contrast generated by the gold-labelled MSCs by micro-CT regardless of whether they were delivered SC or IV. Silva et al. also reported failure to detect labelled cells in vivo by micro-CT [38], and of the studies reported in Table 1, this is the only one with negative results. They labelled hMSCs with dimercaptosuccinic acid (DMSA) gold nanoparticles (GNPs) for 24 h at a concentration of 90 μg/mL and observed a slightly higher contrast than unlabelled MSCs in micro-CT phantoms, but the difference was not significant, and intranasal inoculation of labelled MSCs did not result in detectable contrast by in vivo micro-CT.

Here, although not highly toxic, the gold concentration used showed reduced cell viability after 24 h. The fact that other groups have used higher labelling concentrations might be explained as GNP toxicity depends on functionalisation and uptake [39]. The effects of surface modifications have been studied in various cell lines. Naked GNRs negatively affect mammalian cells at concentrations as low as 0.7 μg/mL whilst silica coating increases the cell tolerance to GNRs [40] consistent with the viability of hUC-MSCs exposed to the silica-coated GNRs in this study.

Silica shell thickness plays a key role in preventing plasmon coupling and preserving the optical signature of the GNRs after cell uptake, which are important considerations for optimal MSOT imaging [15]. Whilst the commercial GNRs used here lost their silica coating during labelling (contrarily to those used in [15]), MSOT still enabled the detection of the GNR-labelled hUC-MSCs in the mouse flanks. In contrast to MSOT, aggregation might work in favour of the detection of gold by micro-CT by preventing the GNRs from being removed by exocytosis which would reduce intracellular gold [29, 41, 42]. Despite this aggregation phenomenon potentially taking place in our study, the GNR hUC-MSCs were still undetectable by micro-CT.

High gold uptake per cell is necessary to achieve good contrast as micro-CT signal increases proportionally with increasing gold concentrations; however, cell uptake usually reaches saturation [43, 44]. The GNRs used here had an average core size of 56 nm × 18 nm, which results in high uptake by receptor-mediated internalisation [45]. We used the highest concentration that did not induce overt toxicity. Despite this, no micro-CT signal was detected in our study. When we imaged nanoparticle suspensions via micro-CT we only observed contrast at 80 mM and even at this concentration, the contrast generation was small (Figure S1). This concentration is 60-fold higher than the concentration we used to label the cells and it is therefore very unlikely that sufficient intracellular levels of gold to generate micro-CT contrast would have been obtained in our experimental set-up.

The discrepancy between our results and those of other studies raises the question of the limit of detection of micro-CT for gold. This determination is not straightforward as micro-CT scanning conditions along with the properties of the gold nanoparticles impact x-ray attenuation [46].

Attempts at determining the minimal amount of gold necessary to achieve contrast in micro-CT have been made using phantom imaging. Galper et al. established that the attenuation of gold is 5.1 HU/mM [46]. This is a physical parameter that should not vary between research groups. We attempted to evaluate the attenuation of gold corresponding to results in the publications reporting cell tracking with micro-CT. Table 2 shows those estimated HU/mM attenuations. To arrive at these numbers, we calculated the molar concentration of gold in the micro-CT phantoms used in the studies using Equation (1):

(1) $$\frac{\left(\frac{\left(\text{Au}\;\text{per}\;\text{cell}\;[\text{g]}\right)}{197}\times \;\text{cell}\;\text{number}\;\right)}{\;\text{cell}\;\text{suspension}\;\text{volume}\;(\text{L})}=\;\text{Au}\;\text{concentration}\;(\text{M})$$

It is noteworthy that most studies show a much higher HU/mM attenuation in their phantom studies when compared to Galper’s data. Considering the 5.1 HU/mM attenuation, Cormode and colleagues concluded that 5.8 mM is the minimum detectable gold concentration [47]. Considering a cell volume of 8 pL, the minimum amount of gold per cell necessary to achieve a 5.8 mM (1.16 g/L) concentration for a voxel filled entirely with labelled cells is 9 pg of gold/cell (Equation (2)):

(2) $$\begin{array}{l} \text{Cell}\;\text{volume}\times \text{Au}\;\text{concentration}=\;\text{Minamount}\;\text{of}\;\text{Au}\;\text{per}\;\text{cell} \end{array}$$

The gold/cell column in Table 2 shows that all studies except for Silva et al. [38] achieved a nanoparticle load per cell higher than the 9 pg detection threshold, potentially explaining why Silva’s study is the only one that failed to visualise the gold-labelled cells by micro-CT. It is thus clear that extremely high cellular uptake of GNRs is necessary in order to obtain micro-CT contrast, which increases costs and may adversely affect cell health. It is worth mentioning that the limits of detection of gold particle-labelled cells is partly due to the poor lateral resolution of micro-CT, which averages the signal over the whole cells, rather than focussing on the endo-lysosomal compartment where the particles are located. With modern x-ray optics such as the optics available in synchrotrons, it is possible to generate nano-focused X-ray beams that enable lower particle concentrations to be detected [48].

The main limitation of our study is that we did not quantify the amount of gold per cell. Given the lack of contrast observed during the imaging of cell phantoms as well as in vivo, it is clear that even at the highest labelling concentration, the GNRs did not accumulate in high enough numbers inside the cells and thus, were not detectable by micro-CT. On the contrary, they were easily detectable in the same conditions by MSOT when injected subcutaneously, showing that this imaging modality is significantly more sensitive than micro-CT.

## 5 Conclusion

We tested the feasibility of a non-invasive, multi-modal imaging approach that utilises a combination of GNRs and reporter genes to track MSCs after subcutaneous or intravenous injection in vivo. This labelling approach did not affect cell morphology and viability of hUC-MSCs significantly and allowed for robust tracking of the cells by BLI for both IV and SC delivery. The GNR-labelled cells were detectable by MSOT when injected subcutaneously validating the ability of a BLI/MSOT tracking approach. Although micro-CT produces anatomical images of the lungs, the same GNR-labelled cells could not be detected within this organ or in the flanks of the mice indicating that the cells did not carry enough contrast agent to be tracked by micro-CT.

To provide enough contrast for micro-CT imaging, large amounts of gold are necessary. However, high labelling concentrations might impair cell viability making micro-CT tracking of gold-labelled cells challenging.

In summary, our study found that multi-modal imaging of MSCs labelled with gold nanorods and the reporter gene firefly luciferase allows BLI and MSOT detection of administered cells in vivo; however micro-CT lacks sensitivity towards gold under the conditions investigated.

## Figures and Tables

**Table 1:** Overview of articles reporting pre-clinical micro-CT tracking of gold labelled cells.

Cell type	Cell number	Animal model	Administration route	Tracking time	Labelling conditions	GNP characteristics^a^	Gold/cell	Imaging	Cells detected?	Ref
hUCMSCs	4 × 10^6^	Mouse IPF	Tracheal infusion	1, 9, 25, 35 days	100 μg/mL, 4 h	127.3 nm, CPP-PSD, pH-sensitive	313.5 pg	In vivo	Y	[27]
hUCMSCs	4 × 10^6^	Mouse IPF	Tracheal infusion	1, 4, 7, 10 days	1000 μg/mL, ≥ 12 h	40 nm, PEG-TAT, RBITC-labelled	920 pg	In vivo	Y	[28]
hUCMSCs	4 × 10^6^	Mouse IPF	Tracheal infusion	1,3,5,7,10 days	200 μg/mL, 12 h	40 nm, temperature responsive	120 pg	In vivo	Y	[29]
hUCMSCs	1.5 × 10^6^	Mouse IPF	Tracheal infusion	3,48 h, 9, 16, 23 days	200 μg/mL, 24 h	10.7 ± 1.7 nm, BSA-PLL	293 pg	In vivo	Y	[30]
Rat glioma C6 cell line	1 × 10^5^	Wistar rat	Stereotactic injection into brain	16 days, ex vivo	50 μg/mL, 22 h	50 nm, colloidal	0.04 ng	Ex vivo	Y	[31]
hUCMSCs	2 × 10^5^	RCS rat model	Subretinal injection	1, 15, 30 days post injection	1.4 × 10^8^ particles /mL, 24 h	80 nm, colloidal	Not mentioned	In vivo	Y	[32]
Human periodontal ligament stem cells (hPDLSCs)	1 × 10^6^	Wistar rats	- Intramuscular - Subcutaneous - Submucosal - Subgingival	0, 2, 5 days	50 μg/mL, 12 h	40 nm, PLL-hydrobromide, RBITC-labelled	Not mentioned	In vivo	Y	[33]
hMSCs (undetermined source)	2 × 10^4^ or 5 × 10^5^	Sprague Dawley rats	Stereotactic injection into brain	30 min post injection	100 μg/mL, 12 h	40 nm, PLL, RITC- labelled	382.5 pg	In vivo	Y	[34]
Bone marrow MSCs	1 × 10^6^	BL/6 IPF	Tracheal infusion	7, 14, 21 days	200 μg/mL, 24 h	12.2 nm ± 1.59 nm, Albumin-PLL, ICG-labelled	218 pg	In vivo	Y	[35]
hMSCs (undetermined source)	1 × 10^6^	Nude mice	Intra-arterial (carotid artery)	24 h	52 μg/mL, 22 h	50 nm, colloidal	332 ± 2 μg	Post mortem	Y	[36]
F98 rat glioma cells	1 × 10^5^ or 2 × 10^5^	Nu/nu mice	Stereotactic injection into brain	6–8 days after tumour implantation	No dose mentioned, 4 h	50 nm, colloidal	26 500 GNPs	In vivo	Y	[37]
Dental pulp MSCs	5 × 10^5^ and 1 × 10^6^	Mouse dilicosis	Intranasal	Daily up to 7 days	90 μg/mL, 24 h	26.4 ± 0.96 nm, DMSA	4 Pg	In vivo	N	[38]

Abbreviations: CPP, cell penetrating peptide; DMSA, 2,3-dimercaptosuccinic acid; IPF, Idiopathic pulmonary fibrosis; N, no; PEG, polyethylene glycol; PLL, poly-L-lysine; PSD, polymer polysulfonamide; RBITC, rhodamine B isothiocyanate; RCS, Royal college of surgeons; TAT, trans-activator of transcription; Y, yes.

a Gold nanoparticles (GNP) characteristics in order of appearance are size, coupling/coating, other characteristics.

**Table 2:** HU/mM attenuation estimated from the published literature where micro-CT imaging of gold-labelled cell phantoms was undertaken.

Cell type	Cell no.	Cell suspension volume (μL)	Labelling concentration (μg/mL)	Incubation time (h)	GNP characteristics	Gold/cell	HU/mM	Ref
hUCMSCs	1 × 10^6^	30^a^	100	4	127.3 nm pH-sensitive CPP-PSD@Au	313.5 pg	39.9	[27]
hUCMSCs	1 × 10^6^	30^a^	1000	12	40 nm Au@TAT	920	4.2	[28]
hUCMSCs	?	30^a^	200	12	40 nm temperature responsive GNPs	120 pg	20.8	[29]
hUCMSCs	1 × 10^6^	30^a^	200	24	10.7 ± 1.7 nm Au@BSA@PLL	293 pg	99.3	[30]
hMSCs	1 × 10^6^	50	100	12	40 nm AuNP-PLL-RITC poly-L-lysine	382.5 pg	21.0	[34]
BMSCs	3 × 10^6^	30^a^	200	24	(AA@ICG@PLL)	218 pg	4.2	[35]
Dental pulp MSCs	?	30^a^	90	24	Au-DMSA	4 pg	521.2	[38]

*Note*: Question mark (?) indicates that the cell number was assumed to be 1 × 10^6^ cells as this information was not indicated in the papers.

a The cell suspension volume was estimated based on a pellet of 1 × 10^6^ cells.

## Data Availability

All datasets from this study are publicly available on Zenodo, http://doi.org/10.5281/zenodo.6624805 [49].
